# First-time diagnosis of ADHD in adults: challenge to retrospectively assess childhood symptoms of ADHD from long-term memory

**DOI:** 10.1007/s00787-023-02244-2

**Published:** 2023-06-08

**Authors:** Robert Waltereit, Stefan Ehrlich, Veit Roessner

**Affiliations:** 1 Department of Child and Adolescent Psychiatry, LWL-Klinikum Marsberg, Bredelarer Str. 33, 34431 Marsberg, Germany; 2 Department of Child and Adolescent Psychiatry, University Medical Center Göttingen, Von-Siebold-Str. 5, 37075 Göttingen, Germany; 3 Division of Psychological and Social Medicine and Developmental Neurosciences, Translational Developmental Neuroscience Section, Faculty of Medicine, Technische Universität Dresden, Fetscherstrasse 74, 01307 Dresden, Germany; 4 Department of Child and Adolescent Psychiatry, Faculty of Medicine, University Hospital Carl Gustav Carus, TechnischeUniversität Dresden, Fetscherstrasse 74, 01307 Dresden, Germany

Attention-deficit/hyperactivity disorder (ADHD) is an established condition with high prevalence and strong negative impact on mental health, academic and socio-economic performance and quality of life not only in children and adolescents, but also in adults [1–3]. Although once considered a childhood limited disorder, ADHD symptoms and related impairments [4] often persist into adulthood and up to 4.4% of all adults meet criteria for ADHD [5, 6]. Unlike in younger individuals, ADHD is often left untreated in adults. This is due to both the poor adherence to ADHD treatment initiated in childhood or adolescence and the frequent under-diagnosis of ADHD in adults [7, 8].

Speaking of under-diagnosis of ADHD in adults: there are two complex challenges to be faced when diagnosing ADHD in adults for the first time. One is to differentiate the cluster of ADHD symptoms from those of other psychiatric diagnoses that are frequently seen in adults. The other challenge is to reconstruct the presence of ADHD in childhood, as the existence of several ADHD symptoms before the age of 12 years is mandatory to diagnose ADHD as a neurodevelopmental disorder, according to ICD-11 and DSM-5 [9, 10]. Thus, the terms ‘childhood/adolescent/adult ADHD’ and ‘ADHD in childhood/adolescence/adulthood’ all refer to the same entity. The term ‘childhood/adolescence-onset ADHD’ belongs to ‘neurodevelopmental ADHD’, whereas the recently proposed concept of ‘adult-onset ADHD’ may or may not belong to it. It is important to distinguish between cases of ADHD in adults where neither ADHD symptoms nor an ADHD diagnosis have been present in childhood and cases where ADHD symptoms or a diagnosis have been present in the past.

Even if there were really such cases of individuals with first-onset of ADHD symptoms in adulthood, this concept seems to apply to a negligible proportion of adults diagnosed with ADHD [11, 12]. Nevertheless, a lively debate continues to this day about whether “adult (first)-onset ADHD” really exists. In their very interesting review, Taylor et al. [6] state that the inadequate methods of the identified studies currently provide unclear information about the nature of late first-onset ADHD symptoms. While these constellations of symptoms seem to exist in adults “…their source could be (1) adult-emergent symptoms that were previously surpassed due to lower environmental demands/supportive facilitators, (2) mimics that were not properly assessed, or (3) childhood-onset symptoms that were not detected earlier due to failure to come to clinical attention.” In this vein, we consider the discussion somewhat “academically playful” regarding whether and how often ADHD diagnosed for the first time in adulthood is (a) truly first-onset, non-neurodevelopmental ADHD versus (b) a first-time diagnosis of neurodevelopmental ADHD in adulthood based on previously unrecognized ADHD symptoms. This may be seen regardless of the reason why these symptoms were not recognized or were not detectable and did not lead to a diagnosis in the past, although the current symptoms and a more detailed medical history now justify the diagnosis of “ADHD in adulthood” or “adult ADHD”.

As mentioned above, adults who present for a first-time diagnosis of ADHD pose a challenge to clinicians due to poor recall of childhood symptoms [13], including a tendency to over- or under-report their symptoms [6]. We focus here on the question: how can the clinician reliably assess ADHD symptoms in childhood, that may have been present years or even decades ago? How can he or she now give a previously undiagnosed ADHD diagnosis with sufficient certainty based on this information?

In the absence of a biomarker, until today the retrospective diagnosis of childhood ADHD can only be made by clinical assessment. In particular, a clinical-diagnostic interview to assess the individual’s developmental history and the use of appropriate retrospective rating scales are the main methods for this. In all of these methods, the valid retrieval of necessary information from the reviewer's (patients themselves, parents/caregivers and third-party informants) long-term memory is essential. Uncertainty regarding the validity of this information addresses a key question in psychiatric diagnostics; moreover, it addresses questions known also in philosophy, psychology and jurisprudence. To what extent can we trust non-experts’ long-term memory regarding specific professional terms, which may additionally be biased by informants’ subjective views and expectations regarding the advantages or disadvantages of such a diagnosis?

Developmental history, obtained through a clinical diagnostic interview, is a widely used tool to assess academic performance, social behavior and possible problems from pre-school and primary school times. We have published a pilot study about the importance and usefulness of the developmental history in the diagnosis process, even in significant details, in patients with ADHD older than 12 years. We found that parents of those patients retrospectively already reported problematic behaviors in early and middle childhood [14]. The diagnostic value of developmental history regarding ADHD compared to other psychiatric conditions has otherwise, however, been almost unexplored. In particular, there is no scientific evidence for reconstructing symptoms in childhood that would have justified an ADHD diagnosis at that time, based on developmental history, obtained through a clinical diagnostic interview.

Rating scales have been developed to systematically and as validly as possible assess ADHD symptoms in childhood based on informant recall (e.g. patients themselves, parents/caregivers and third-party informants). The short versions of the Wender Utah Rating Scale (WURS), self-rating scales, have gained particular importance in adult psychiatry and were promoted as standard of care, e.g. by the Federal Joint Committee (Gemeinsamer Bundesausschuss, G-BA), a public legal entity comprising the four leading umbrella organizations of the self-governing German healthcare system under the statutory supervision of the German Federal Ministry of Health [15]. In contrast to “the ennoblement by these state honours” resulting in a currently generally recognized de-facto standard in German adult psychiatry, the diagnostic validity of the WURS and its short forms has been and continues to be questioned [16–19]. The main reason for that is that retrospectively rating ADHD symptoms in childhood solely based on long-term recall of the patient has been criticized as being of poor validity in the majority of cases [20].

In many European countries, school reports are available which, unlike subjective recall with the high risk of bias, still reflect the evaluation by teachers at that time unchanged even after decades. School reports are routinely written by teachers at the end of a term. They contain detailed information about academic performance and social behavior-related to symptoms of ADHD, notably inattention. Consequently, clinical guidelines recommend their use for the assessment of childhood ADHD [21, 22]. An important research gap, with corresponding clinical implications, still exists today in that school reports, despite their intensive use in clinical practice, have not been scientifically evaluated for their validity in retrospectively diagnosing childhood ADHD. In addition, there is no systematic rule how to assess school reports for this purpose. Every clinician does it at his/her own convenience.

Taken together, the management of a key diagnostic issue in the first-time diagnosis of ADHD in adolescents and particularly in adults is currently unsatisfactory, as there is neither a scientific basis nor a certain established guideline in this regard. Presumably, this is part of the explanation for why there is often under-diagnosis but also potentially over-diagnosis of adult ADHD. This may lead to wrong treatment of false-negative or false-positive ADHD diagnoses in certain situations. We suggest that novel ideas and scientific resources should be invested here to bring fresh thinking to this currently overlooked or neglected clinical problem.

## References

[1] Coghill D, Banaschewski T, Cortese S (2021). The management of ADHD in children and adolescents: bringing evidence to the clinic: perspective from the European ADHD guidelines group (EAGG). Eur Child Adolesc Psychiatr.

[2] Overgaard KR, Oerbeck B, Friis S (2022). Attention-deficit/hyperactivity disorder from preschool to school age: change and stability of parent and teacher reports. Eur Child Adolesc Psychiatr.

[3] Widding-Havneraas T, Markussen S, Elwert F (2022). Geographical variation in ADHD: do diagnoses reflect symptom levels?. Eur Child Adolesc Psychiatry.

[4] Li J, Wang W, Cheng J (2022). Relationships between sensory integration and the core symptoms of attention-deficit/hyperactivity disorder: the mediating effect of executive function. Eur Child Adolesc Psychiatr.

[5] Breuer D, von Wirth E, Mandler J (2022). Predicting delinquent behavior in young adults with a childhood diagnosis of ADHD: results from the cologne adaptive multimodal treatment (CAMT) study. Eur Child Adolesc Psychiatr.

[6] Taylor LE, Kaplan-Kahn EA, Lighthall RA, Antshel KM (2022). Adult-onset ADHD: a critical analysis and alternative explanations. Child Psychiatr Hum Dev.

[7] Luo X, Guo X, Zhao Q (2022). A randomized controlled study of remote computerized cognitive, neurofeedback, and combined training in the treatment of children with attention-deficit/hyperactivity disorder. Eur Child Adolesc Psychiatr.

[8] Bachmann CJ, Philipsen A, Hoffmann F (2017). ADHS in Deutschland: trends in diagnose und medikamentöser therapie. Dtsch Arztebl Int.

[9] American Psychiatric Association (2013). DSM-V. Diagnostic and statistical manual of mental disorders.

[10] World Health Organization (2022) ICD-11: International classification of diseases (11th revision). https://icd.who.int/

[11] Sibley MH, Rohde LA, Swanson JM (2018). Late-onset ADHD reconsidered with comprehensive repeated assessments between ages 10 and 25. Am J Psychiatr.

[12] Solanto MV (2019). The prevalence of “late-onset” ADHD in a clinically referred adult sample. J Atten Disord.

[13] Katzman MA, Bilkey TS, Chokka PR (2017). Adult ADHD and comorbid disorders: clinical implications of a dimensional approach. BMC Psychiatr.

[14] Waltereit J, Haas F, Ehrlich S (2019). Family and developmental history of ADHD patients: a structured clinical routine interview identifies a significant profile. Eur Arch Psychiatr Clin Neurosci.

[15] Gemeinsamer Bundesausschuss (G-BA) (2013) Arzneimittel-Richtlinie/ Anlage III: Nummer 44—Stimulantien. https://www.g-ba.de/beschluesse/1677/

[16] Hill BD, Pella RD, Singh AN (2009). The Wender Utah Rating Scale: adult ADHD diagnostic tool or personality index?. J Atten Disord.

[17] McCann BS, Scheele L, Ward N, Roy-Byrne P (2000). Discriminant validity of the Wender Utah Rating Scale for attention-deficit/hyperactivity disorder in adults. J Neuropsychiatr Clin Neurosci.

[18] Suhr J, Zimak E, Buelow M, Fox L (2009). Self-reported childhood attention-deficit/hyperactivity disorder symptoms are not specific to the disorder. Compr Psychiatr.

[19] Wolf F, Heinzel-Gutenbrunner M, Becker K (2018). Retrospective recording of childhood ADHD symptoms: follow-up of adults formerly diagnosed with childhood ADHD and/or childhood conduct disorder. Nervenarzt.

[20] Mannuzza S, Klein RG, Klein DF (2002). Accuracy of adult recall of childhood attention deficit hyperactivity disorder. Am J Psychiatr.

[21] AWMF (Arbeitsgemeinschaft der Wissenschaftlichen Medizinischen (2018) Langfassung der interdisziplinären evidenz-und konsensbasierten (S3) Leitlinie „Aufmerksamkeitsdefizit‑/Hyperaktivitätsstörung (ADHS) im Kindes‑, Jugend- und Erwachsenenalter“. AWMF-Registernummer 028–045

[22] National Institute for Health and Care Excellence (2018) Clinical guideline 87: Attention deficit hyperactivity disorder: diagnosis and management of ADHD in children, young people and adults. https://www.nice.org.uk/guidance/NG87 29634174

